# A Turn-On Fluorescent Sensor for Glutathione Based on Bovine Serum Albumin-Stabilized Gold Nanoclusters

**DOI:** 10.1155/2018/1979684

**Published:** 2018-12-02

**Authors:** Yan Qiu, Jianlin Huang, Li Jia

**Affiliations:** ^1^Ministry-of-Education Key Laboratory for the Synthesis and Application of Organic Functional Molecules & College of Chemistry and Chemical Engineering, Hubei University, Wuhan 430062, China; ^2^Yunnan Branch of China National Geological Center of Building Materials Industry, Kunming 650118, China

## Abstract

A fluorescence sensor for the detection of glutathione based on the fluorescence recovering of the bovine serum albumin-stabilized gold nanoclusters is reported. This study indicates that glutathione restores the copper-ion-quenched fluorescence by coordinating the bound copper ion in the bovine serum albumin molecule used for stabilizing the gold nanocluster through its sulfydryl. Under the experimental conditions, the fluorescence response showed a linear relationship with the concentration of glutathione over the range from 10 *µ*M to 400 *µ*M. The fluorescence sensor successfully detected glutathione in commercial drug products.

## 1. Introduction

Development of glutathione (GSH) assay methods has received attention due to its diverse functions in organisms and extensive market prospects. GSH, an important nonenzymatic antioxidant, is found in almost all cell types. GSH protects cells from damage of reactive oxygen species such as hydroxyl radical, hydrogen peroxide, and lipid peroxides, directly by eliminating free radicals, and indirectly by serving as a cofactor for glutathione peroxidase [1, 2]. GSH also participates in other physiological processes such as control of cell proliferation and nucleotide metabolism [3, 4]. Based on its essential role in the health of organisms, GSH is used in clinic to treat kinds of diseases such as liver disease and uremia and reduce the side effects correlated with chemoradiotherapy.

Many analytical methods, such as high performance liquid chromatography, capillary electrophoresis, fluorophotometry, and electrochemistry, have been developed for detection of GSH [5–8]. Among these methods, fluorophotometry has advantages over the other techniques at sensitivity, simplicity, and costs. In recent years, fluorescent probes for detection of GSH have been designed and investigated for overcoming the disadvantages of traditional fluorometric assays [9–12]. Although these fluorescent probes successfully detected GSH from various samples, including aqueous solutions, human serum, bovine serum album (BSA), and liposome, they suffered from complicated and tedious synthesis procedures.

Bovine serum albumin-protected fluorescent gold nanoclusters (AuNCs-BSA) reported by Xie et al. have given rise to research interest in sensing applications owing to the advantages of facile preparation, high fluorescence quantum yield (~6%), favorable photostability, and good biocompatibility [13]. Xie's research group developed a simple label-free method for the selective and sensitive detection of Hg^2+^ based on fluorescence quenching of AuNCs-BSA triggered by Hg^2+^-Au^+^ interactions [14]. Liu et al. reported a AuNCs-BSA-based fluorescent sensor for the recognition and determination of cyanide in aqueous solution, which was based on the fluorescence quenching of AuNCs-BSA induced by the Elsner reaction between cyanide and gold atoms of AuNCs-BSA [15]. Durgads et al. demonstrated the AuNCs-BSA can be used as a selective fluorescence “turn-off” sensor for Cu^2+^ in live cells based on fluorescence quenching of AuNCs-BSA resulting from intersystem crossing of the excited electron from the gold cluster stimulated by the bound Cu^2+^ in the BSA molecule [16]. Their paper also showed that the copper-ion-quenched emission was reversible with a copper chelator glycine.

A previous study demonstrated that the fluorescence of GSH-capped gold nanoparticles was quenched by Cu^2+^ due to the complexation between Cu^2+^ and GSH [17]. Thus, we assumed that GSH might be able to retrieve the copper-ion-quenched fluorescence of AuNCs-BSA by coordinating Cu^2+^. GSH was found to be much more effective than glycine on restoring the fluorescence quenched by copper ions in our study. Thus, we have developed a fluorescence “turn-on” sensor for GSH based on the AuNCs-BSA-Cu system.

## 2. Materials and Methods

### 2.1. Reagents

Glutathione (98%), HEPES (99%), and amino acids (≥98%) were purchased from Aladdin Biochemical Technology Co., Ltd. (Shanghai, China). HAuCl_4_ (AR), bovine serum albumin (BR), NaOH (AR), and metal nitrates (AR) were purchased from Sinopharm Chemical Reagent Co., Ltd. (Shanghai, China). Reduced glutathione for injection and reduced glutathione tablets were from YaoPharma Co., Ltd. (Chongqing, China). Ultrapure water with 18.2 MΩ·cm resistivity was used for preparing the solutions.

### 2.2. Preparation of AuNCs-BSA

The AuNCs-BSA was synthesized based on a modified Xie method [13]. In brief, HAuCl_4_ solution (25 mL, 10 mM) was mixed with BSA solution (25 mL, 30 mg·mL^−1^) under vigorous stirring at 37°C. Two minutes later, NaOH solution (3 mL, 1 M) was introduced, and the mixture was incubated at 37°C for 12 h.

### 2.3. Detection of GSH

For fluorescent detection of GSH, varying volumes of 10 mM GSH solutions were mixed with the AuNCs-BSA solution containing Cu^2+^ which was prepared by adding 30 *µ*L 10 mM Cu^2+^ solution to 250 *µ*L AuNCs-BSA solution, and the mixtures were diluted to 5 mL with HEPES buffer (pH=7.2). Fluorescence emission spectra of the as-prepared solutions were measured under 480 nm excitation.

### 2.4. Sample Preparation

A bottle of reduced glutathione powder for injection was dissolved and diluted to 100 mL with ultrapure water. After four reduced glutathione tablets were ground, the powder was dissolved in ultrapure water and filtered. The filtrate was finally diluted to 100 mL with ultrapure water.

## 3. Results and Discussion

### 3.1. Mechanism for Fluorescence Recovering of AuNCs-BSA

**Figure 1:**
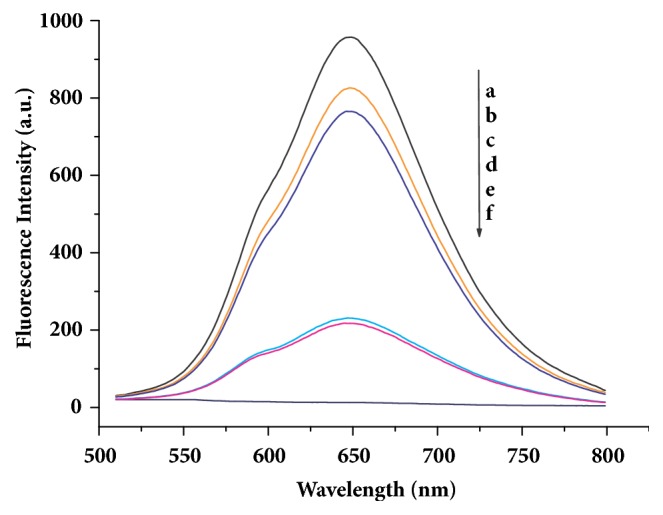
Fluorescence emission spectra of AuNCs-BSA in the absence (a) and presence of Cu^2+^ (f). Fluorescence recovery from the quenched AuNCs-BSA by adding of GSH (b), cysteine (c), glycine (d), and glutamic acid (e).

The deep brown solution of AuNCs-BSA emits a red fluorescence under 480 nm excitation. The fluorescent emission peak at 648 nm was found to disappear upon addition of 300 *µ*M Cu^2+^. It was further observed that the AuNCs-BSA solution containing Cu^2+^ emitted strong fluorescence again after treatment with of 1.6 mM GSH (Figure 1).

The fluorescence quenching of AuNCs-BSA in the presence of Cu^2+^ was attributed to the binding of Cu^2+^ on to the BSA used for stabilizing the gold nanocluster, which enabled the paramagnetic Cu^2+^ to prompt intersystem crossing of the excited electron from the gold cluster and consequently decreased the fluorescence intensity [16]. A control experiment showed that GSH had no influence on the fluorescence spectrum of AuNCs-BSA in the absence of Cu^2+^, indicating that the fluorescence recovery induced by adding GSH to the AuNCs-BSA-Cu system resulted from the interaction between GSH and Cu^2+^. GSH, a natural tripeptide that consists of glutamate, cysteine, and glycine, contains various coordinating function groups such as carboxyl, amido, sulfydryl, and acylamino, which facilitates its molecules to form complexes with metal ions. GSH was replaced by glutamic acid, cysteine, and glycine, respectively, to observe the change in fluorescence properties of the AuNCs-BSA-Cu system and identify the binding site on GSH for Cu^2+^. It is apparent in Figure 1 that the fluorescence intensity restored by cysteine was close to that by GSH at the same concentration and much stronger than that by glycine or glutamic acid. Considering the facts that Cu^2+^ is characterized by a strong affinity for SH residues and among the three amino acids constituting GSH only cysteine has a sulfydryl, we speculate that GSH recovers the copper-quenched fluorescence of AuNCs-BSA by coordinating the bound Cu^2+^ in the BSA molecule used for stabilizing the gold nanocluster through its sulfydryl.

### 3.2. Optimization of Conditions for GSH Sensing

Concentration dependent effects of AuNCs-BSA and Cu^2+^ on the detection of GSH were investigated. High concentrations of Cu^2+^ were required for high fluorescence quenching efficiency at high concentrations of AuNCs-BSA, which means low detection sensitivity for GSH. On the other hand, too low a concentration of Cu^2+^ would increase background fluorescence and narrow the allowing quantitative range of GSH due to low fluorescence quenching ability. In a solution with a total volume of 5 mL, 250 *µ*L AuNCs-BSA and 60 *µ*M Cu^2+^ were finally selected for GSH sensing.

**Figure 2:**
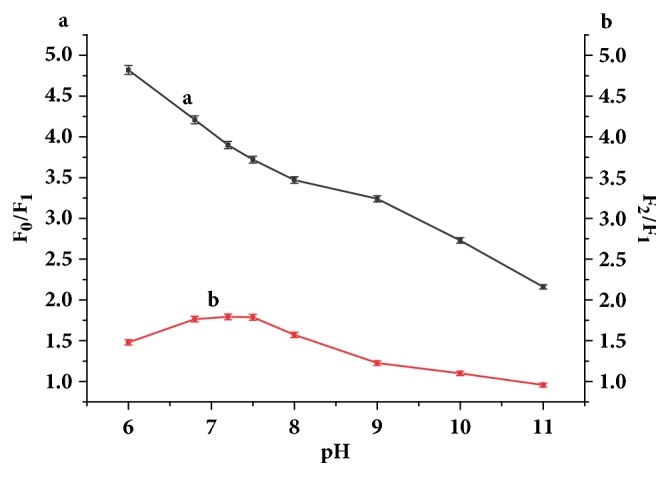
Fluorescence quenching efficiency of Cu^2+^ (a) and fluorescence recovering efficiency of GSH (b) at different pH values.

The acid effect on the sensing system was studied over a pH range from 6 to 11. When the pH value increased in the tested range, diminutive change in the fluorescence intensity of AuNCs-BSA was observed, whereas the fluorescence intensity of the AuNCs-BSA in the presence of Cu^2+^ increased, indicating the fluorescence quenching efficiency of Cu^2+^ decreased with increasing of the pH value. It was also observed that the fluorescence recovering efficiency of GSH changed with the pH value. The fluorescence quenching and recovering efficiency are represented with F_0_/F_1_ and F_2_/F_1_ respectively, where F_0_ and F_1_ correspond to the fluorescence intensity of the AuNCs-BSA in the absence and presence of Cu^2+^, respectively. F_2_ represents the fluorescence intensity of the AuNCs-BSA in the presence of Cu^2+^ and GSH. As shown in Figure 2, the fluorescence recovering efficiency of GSH is stabilized and maximized at physiological pH. The HEPES buffer solution was finally employed to adjust the pH of solutions used in the measurement to 7.2.

**Figure 3:**
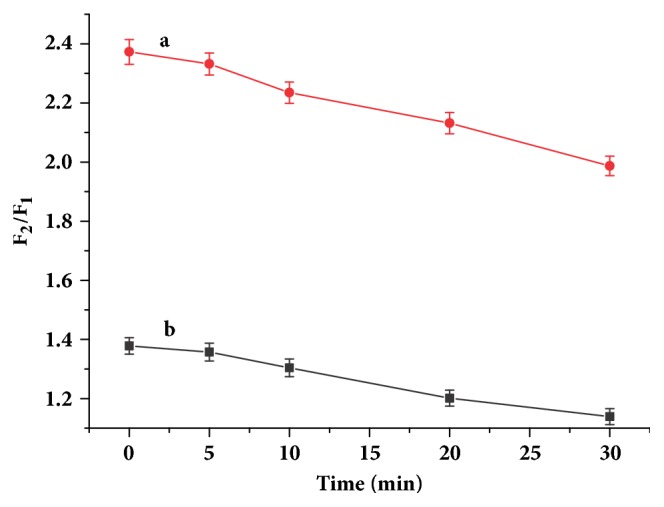
Time-dependent fluorescence recovering efficiency of 200 *µ*M GSH (a) and 40 *µ*M GSH (b).

Time-dependent fluorescence signals of the sensing system were observed. The change in fluorescence properties of AuNCs-BSA in the absence and presence of Cu^2+^ was not obvious within 30 minutes. However, the fluorescence intensity of the AuNCs-BSA in the presence of Cu^2+^ and GSH slowly decreased with time, and thus the fluorescence recovering efficiency decreased with time (Figure 3). Therefore, the fluorescence of the sensing system should be measured immediately upon adding GSH to the solution of AuNCs-BSA in the presence of Cu^2+^.

### 3.3. Selectivity and Sensitivity for GSH Sensing

**Figure 4:**
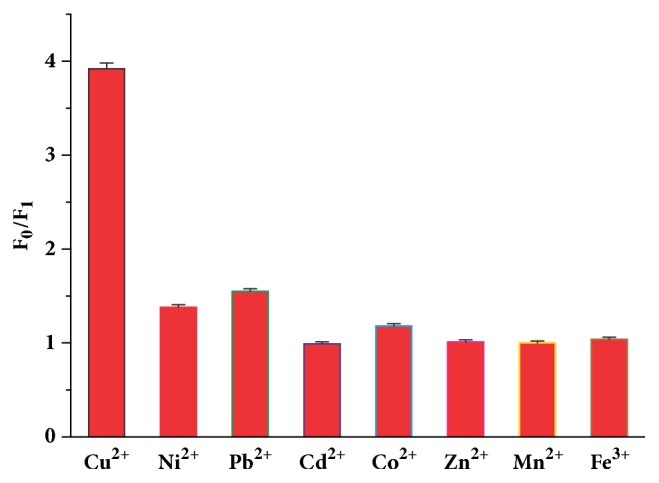
Fluorescence quenching effect of metal ions (F_0_ and F_1_ are the fluorescence intensity of the AuNCs-BSA in the absence and presence of metal ions, respectively.).

**Figure 5:**
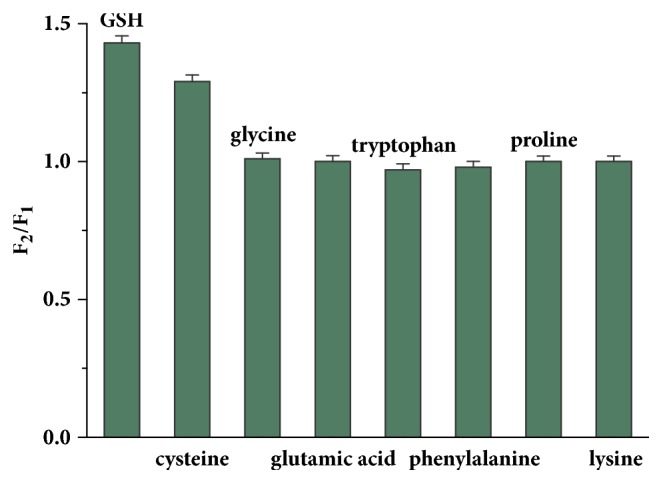
Selectivity of the sensor for GSH over amino acids (F_2_ is the fluorescence intensity of the AuNCs-BSA in the presence of Cu^2+^ and GSH or amino acids.).

Although the presence of Pb^2+^, Co^2+^, or Ni^2+^ with the same concentration of Cu^2+^ (60 *µ*M) showed a quenching effect on the fluorescence of the AuNCs-BSA, their quenching efficiencies were much lower than that of Cu^2+^ (Figure 4). The degree of interference of other metal ions, including K^+^, Ca^2+^, Mg^2+^, Zn^2+^, Cd^2+^, Mn^2+^, and Fe^3+^, for the detection of GSH was further investigated. On the basis of a relative error range from -5% to 5% in detecting 50 *µ*M GSH, the tolerance concentrations were as follows: 1 mM for K^+^, Ca^2+^, Mg^2+^, 500 *µ*M for Zn^2+^, Mn^2+^, Cd^2+^, and 100 *µ*M for Fe^3+^. Some amino acids were also used to evaluate the selectivity of the sensing system. As shown in Figure 5, only cysteine could result in significant fluorescence recovery of the AuNCs-BSA, whereas no obvious changes in the quenched fluorescence were observed in the presence of other amino acids such as glycine, lysine, proline, glutamic acid, tryptophan, and phenylalanine at the same concentration of GSH (50 *µ*M).

**Figure 6:**
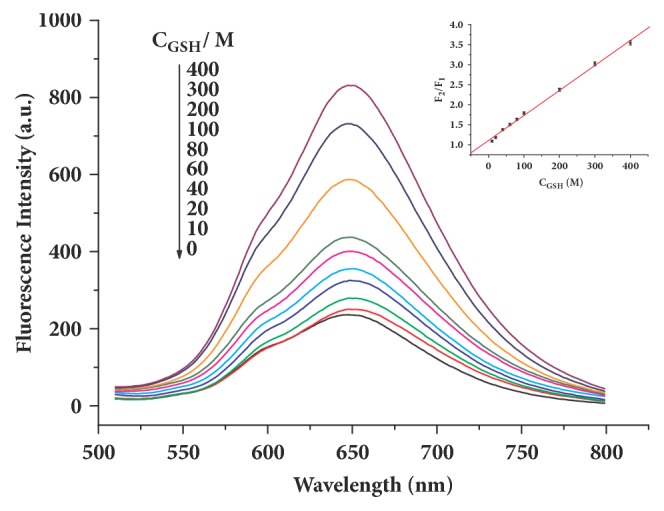
Fluorescence recovery from the quenched AuNCs-BSA by adding of various concentrations of GSH. Inset: relationship between the fluorescence recovering efficiency and the concentration of GSH.

Under the optimum detection conditions, the relationship between the fluorescence recovering efficiency (F_2_/F_1_) and the concentration of GSH over the range from 10 *µ*M to 400 *µ*M could be expressed by a linear equation (R^2^ = 0.996), F_2_/F_1_ = 0.0063C_GSH_ + 1.09 (Figure 6). The limit of detection for GSH was calculated to be 1.2 *µ*M.

### 3.4. Application

Commercial reduced glutathione tablets and reduced glutathione powder for injection were employed as practical samples to evaluate the applicability of the GSH sensor developed here. The recovery and relative standard deviation obtained with a standard addition method through five parallel tests are presented in Table 1.

## 4. Conclusions

We found that GSH restored effectively the copper-quenched fluorescence from the AuNCs-BSA and therefore develop a new fluorescence “turn-on” sensor for GSH detection. The sensor shows advantages such as fast and sensitive response to GSH, simplicity in preparation and usage, and environmental friendliness. The recovery and precision obtained from commercial GSH drug products indicate the potential application of the GSH sensor.

## Figures and Tables

**Table 1:** Determination of GSH in practical samples (n=5).

Samples	Detected/*µ*M	Added/*µ*M	Total found/*µ*M	Recovery/%	RSD/%
GSH tablets	155.1	80.0	231.6	98.5	3.9
GSH powder	96.6	50.0	148.5	101.3	2.6

## Data Availability

The data used to support the findings of this study are available from the corresponding author upon request.
